# GXP: Analyze and Plot Plant Omics Data in Web Browsers

**DOI:** 10.3390/plants11060745

**Published:** 2022-03-11

**Authors:** Constantin Eiteneuer, David Velasco, Joseph Atemia, Dan Wang, Rainer Schwacke, Vanessa Wahl, Andrea Schrader, Julia J. Reimer, Sven Fahrner, Roland Pieruschka, Ulrich Schurr, Björn Usadel, Asis Hallab

**Affiliations:** 1 IBG-2 Plant Sciences, Forschungszentrum Jülich, 52428 Jülich, Germany; c.eiteneuer@fz-juelich.de (C.E.); d.wang@fz-juelich.de (D.W.); s.fahrner@fz-juelich.de (S.F.); r.pieruschka@fz-juelich.de (R.P.); u.schurr@fz-juelich.de (U.S.); 2 Faculty of Natural Sciences, Norges Teknisk-Naturvitenskapelige Universitet, 7034 Trondheim, Norway; davidve@stud.ntnu.no; 3 IBG-4 Bioinformatics, Forschungszentrum Jülich, 52428 Jülich, Germany; j.atemia@fz-juelich.de (J.A.); r.schwacke@fz-juelich.de (R.S.); b.usadel@fz-juelich.de (B.U.); 4 Max Planck Institute for Molecular Plant Physiology, 14476 Potsdam, Germany; vwahl@mpimp-golm.mpg.de; 5 Institute for Biology I, RWTH Aachen University, 52062 Aachen, Germany; schrader@bio1.rwth-aachen.de (A.S.); julia.reimer@hs-emden-leer.de (J.J.R.); 6 Faculty of Technology, University of Applied Science Emden/Leer, Molecular Biosciences, 26723 Emden, Germany

**Keywords:** RNA sequencing, metabolomics, data visualization, overrepresentation analysis, correlation, cluster analysis, principal component analysis, scientific plotting, Mapman, Mercator

## Abstract

Next-generation sequencing and metabolomics have become very cost and work efficient and are integrated into an ever-growing number of life science research projects. Typically, established software pipelines analyze raw data and produce quantitative data informing about gene expression or concentrations of metabolites. These results need to be visualized and further analyzed in order to support scientific hypothesis building and identification of underlying biological patterns. Some of these tools already exist, but require installation or manual programming. We developed “Gene Expression Plotter” (GXP), an RNAseq and Metabolomics data visualization and analysis tool entirely running in the user’s web browser, thus not needing any custom installation, manual programming or uploading of confidential data to third party servers. Consequently, upon receiving the bioinformatic raw data analysis of RNAseq or other omics results, GXP immediately enables the user to interact with the data according to biological questions by performing knowledge-driven, in-depth data analyses and candidate identification via visualization and data exploration. Thereby, GXP can support and accelerate complex interdisciplinary omics projects and downstream analyses. GXP offers an easy way to publish data, plots, and analysis results either as a simple exported file or as a custom website. GXP is freely available on GitHub (see introduction)

## 1. Introduction

Modern life science research projects often produce quantitative data, for example to quantify gene expression or metabolite concentration in tissue samples. Many well-established tools exist that carry out the wet lab and bioinformatics procedures to produce such count data from the samples. Not rarely, these pipelines are carried out by third party laboratories. In the subsequent step, the life scientist, having ordered such RNAseq or other Omics experiments, needs to investigate these count data to form scientific hypotheses and identify underlying biological patterns. Typically, this involves plotting the quantitative data, carrying out Principal Component Analyses and correlation-based hierarchical clustering to elucidate differences between experimental conditions. Genetic or metabolic responses to the tested experimental conditions and treatments often are summarized by identification of enriched traits within significantly up- or down-regulated genes or metabolites of interest. These steps often require manual programming, installation of software, or sending potentially confidential data to webservers for analysis. Gene Expression Plotter (GXP) minimizes these requirements by enabling the user to load count data, generate a variety of informative plots, and carry out typical clustering, principal component and overrepresentation analyses, without the need to write any code, install any software, or send the data to third party servers. Furthermore, GXP enables the user to save all loaded data along with the work done, including generated plots and carried out analysis. With this feature the user can not only save the current work to continue at a later time, but also share data, plots, and analysis results with others, simply by sending the exported GXP database file. Naturally, such a file can be published for example in the form of an article’s supplement thus enabling readers to directly obtain the data, see plots and analysis results and even carry out their own subsequent investigations. 

Gene Expression Plotter consumes two types of input tables, which can be prepared with standard spreadsheet programs, e.g., Microsoft Excel, or can be generated directly by the bioinformaticians producing the quantitative data (for details see results Section 2.1). In short, in the input quantifications table, each row represents quantitative values assessed for a single gene or metabolite, and columns correspond to the different samples for which these quantifications were assessed. In addition to the pure quantifications data table a free format information table can be provided. In it, too, rows correspond to the genes or metabolites, as they appear in the quantifications data table, and columns can provide any free text, categorical, or numeric information the user wants to load into GXP. Such free format information typically comprises knowledge about molecular gene function, for example in the form of Mapman4 Bin annotations [1,2,3], InterPro conserved protein domains [4], or Gene Ontology terms [5]. Additionally, in this table, the user can provide information, e.g., in the form of logarithmic fold change values, quantifying how much the expression of a particular gene changes when contrasting two selected experimental conditions, e.g., control versus stress treatment. Or, in case metabolites have already been quantified, information about their respective chemical properties and involved pathways can be provided. Both of this optional categorical and numerical data can later be used in GXP’s enrichment analyses or can be displayed in the captions of generated plots, here comprising free text information, too. Thus, GXP has been developed to consume generic input data, making it a highly versatile tool, particularly in the context of overrepresentation analysis. 

To produce, analyze, visualize, and publish quantitative omics data many tools exist. Among others, they provide means to plot the data, carry out clustering, and conduct principal component and overrepresentation analyses. A number of these tools specialize in RNAseq analysis [6,7,8,9,10,11,12,13,14,15,16,17,18,19,20,21], most of which consume the raw gene expression count data produced by standard gene expression quantifiers [22,23,24,25] and enable the user to identify differentially expressed genes [6,7,8,9,11,12,13,15,16,20,21,26,27] and review the results in form of comprehensive reports and/or plots [6,7,8,9,10,11,12,13,15,16,18,19,20,21,26,27,28]. Some [7,8,9,10,11,12,13,14,18,19,20] are implemented as an R / Shiny [29,30] application or use other forms of graphical user interfaces (GUI) [15,16,21,27]. These GUI tools allow the user (i) to either execute the tool installed locally on their computer and/or use it on a public web-server and (ii), by means of the GUI, eliminate the need to program plots and analyses manually, with few exceptions [6,28] which require some manual coding to make use of the extended provided functionality. By means of integration of curated published data some tools offer specific analyses, e.g., providing high-confidence insights into molecular gene function. One of these, GENAVi [12], enables the identification of differentially expressed genes (DEGs) in human or mouse RNAseq data by contrasting input with published data. OnestopRNAseq [16] is another example and offers several useful analyses by integration of curated public data from several model animal organisms. In addition, Plant Physiospace [31] enables the user to compare differential gene expression data with curated signatures to identify similar genetic responses investigated in other already-published studies. Other tools focus on metabolomics [32,33,34] or, integrating RNAseq and Metabolomics data, the identification of genotype–phenotype relationships [35]. Some of the introduced tools [7,13,28] offer the elucidation of interactive plots where, e.g., hovering with the mouse over data points in a plot summarizing differential gene expression opens another plot illustrating the expression counts of the particular gene represented by the hovered-over data point. For a more detailed review of the above tools, see supplemental Text S1. In comparison with the above tools, GXP has a “downstream focus” on the visualization of quantitative omics data and subsequent clustering, principal component, and overrepresentation analysis. In this context, the key features of GXP are that (i) it does not require manual programming, nor installation of particular software, (ii) it can thus be used on a simple tablet or even smartphone, and (iii) GXP is versatile, in that it can consume any quantitative omics data stemming from RNAseq or metabolomics analyses. This genericity especially includes any additional arbitrary information on the quantified entities, that is, either genes or metabolites. As explained above, this particularly enables the user to carry out overrepresentation analysis on any, numerical or categorical, data the user provides (see results sections “2.1 Handling input and output data” and “2.5 Overrepresentation (enrichment) analysis” for more details). Furthermore, (iv) GXP is the first, immediately accessible, mature implementation of the popular Mapman tool that visually combines quantitative omics data analysis results with diagrams of metabolic pathways and other processes (see results section “2.4 MapMan web browser plots” for more details). Finally, (v) GXP ensures complete data safety. To explain this, consider that even though GXP is deployed on a webserver, once it has been loaded into the user’s browser, it runs there completely independent of that server. This form of implementation is called a “single page application” (SPA) in which the webserver is little more than a file system delivering GXP to the user’s browser. Among other things the implementation as a SPA implies that at no time is any data sent to any server. All analyses are carried out on the user’s computer in the used web-browser and all data remains exactly there. 

Gene Expression Plotter is freely available on GitHub (https://usadellab.github.io/GeneExpressionPlots/; accession date 11 January 2022). Its code has been released as open source (https://github.com/usadellab/GeneExpressionPlots; accession date 11 January 2022) under the GNU public license, version 3. All functions carrying out the above-described analyses are tested with automatic software unit tests to ensure correct calculations. As mentioned, GXP provides means to export all loaded data, generated plots, and carried out analyses results into a single file, dubbed the “GXP database”. Such a GXP database can be used for publication, e.g., in the form of an article supplement. Additionally, GXP can easily be copied and this copy can be published including custom quantitative omics data, plots, and carried-out analyses. Such a copy can be made available, e.g., on GitHub free of charge or any other webserver. The GXP manual has detailed instructions on a simple procedure to set up such a custom copy of GXP and includes screenshots on seven easy steps that only require a GitHub account and a web browser. 

Thus, our new tool, Gene Expression Plotter, enables the end-user to visualize and analyze quantitative data, typically taken from RNAseq or metabolomics analyses, identify similarity between experimental conditions by cluster and principal component analysis, generate visual summaries of genetic or metabolic responses, identify overrepresented transcripts or metabolite characteristics, and even use GXP to publish the data along with plots and analysis results. 

## 2. Results

### 2.1. Handling Input and Output Data

**Figure 1:**
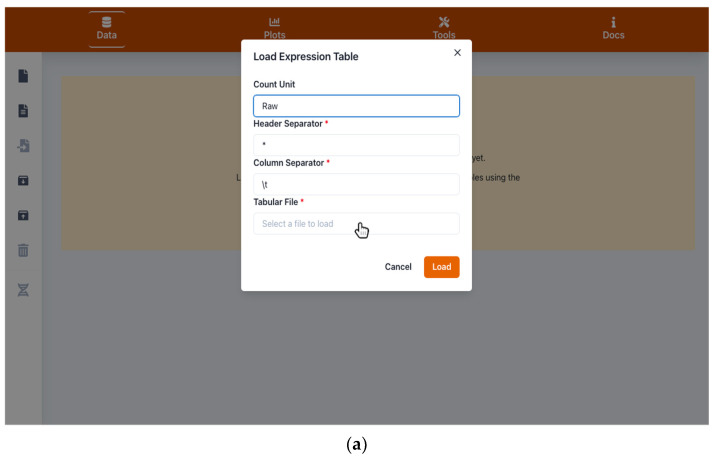

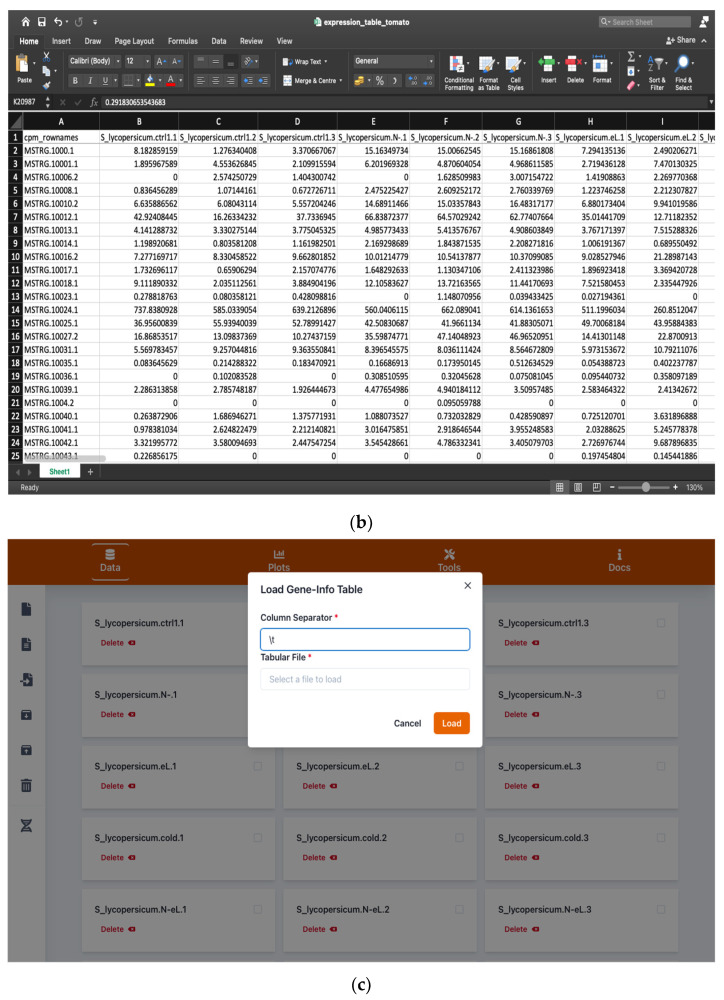

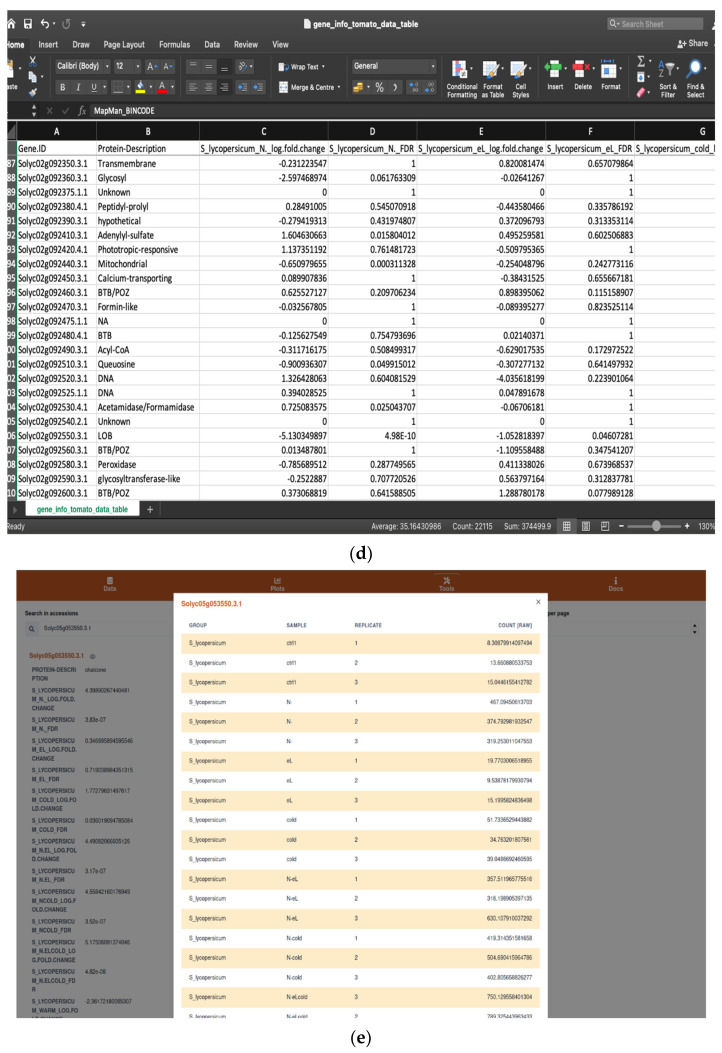

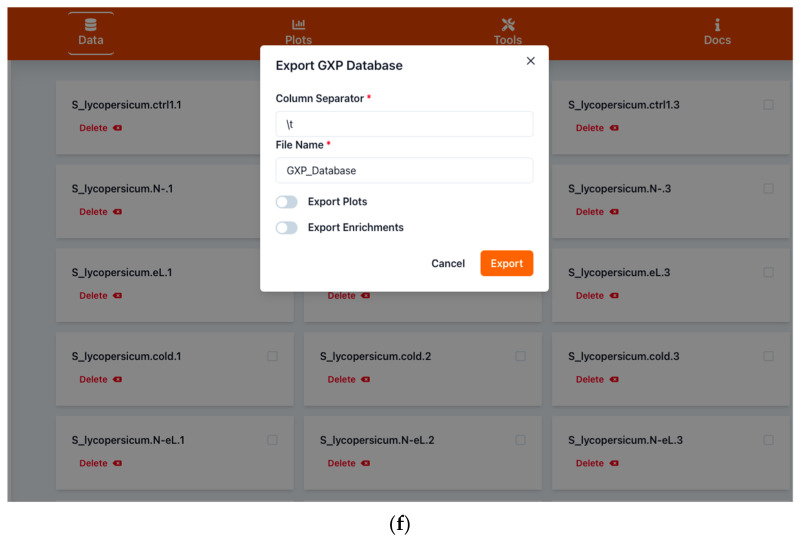
Screenshot images of Gene Expression Plotter (GXP) showing interfaces for data input. (**a**) shows the quantitative data table file import form, triggered by the first document button in the upper left panel. This form is used to load a quantification table such as the one shown in (**b**). (**c**) shows the transcript information table file import form, triggered by the second document icon in the left panel. This form is used to load an optional information table (see Section 1) like the one shown in (**d**). After successful import, the user can search for genes or their annotations in the “gene browser” (helix icon on the lower left panel). In the shown example, the user searched for “chalcone synthase”, a polyketide synthase involved in flavonoid biosynthesis. In (**e**) the user now inspects this gene’s expression quantifications (highlighted foreground) and additional information such as the logarithmic fold changes of gene expression assessed for various comparisons of control and stress treatments (lightly faded out background). Furthermore, as shown in (**f**), by using the GXP export function triggered by the fifth, upward arrow on the box icon in the left panel, GXP enables the user to save the current state, i.e., all imported data, generated plots, and analysis results for later continuation or to share it with other researchers.

**Figure 2:**
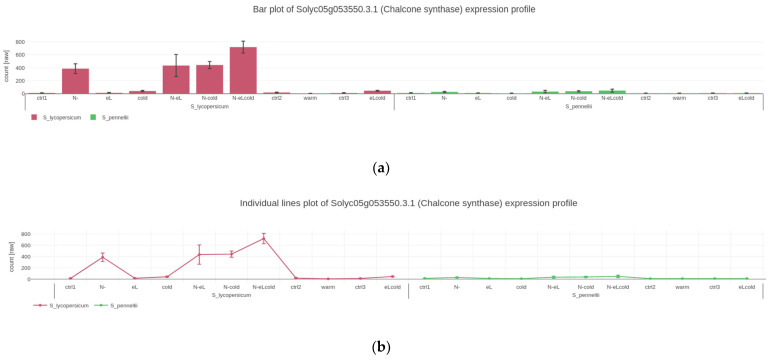

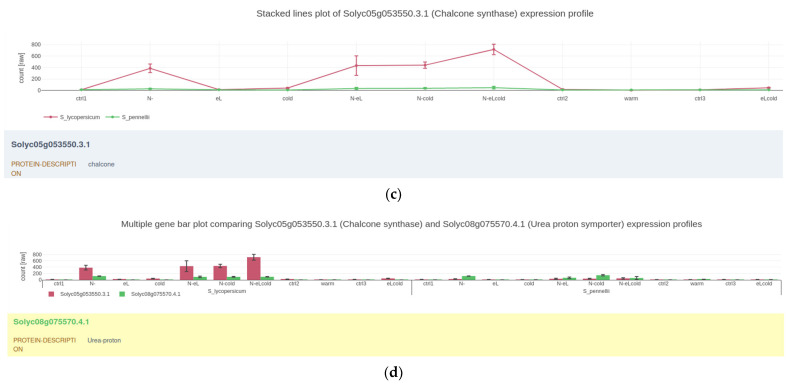
Screenshots of plots visualizing and comparing quantified gene expression between different treatments, experimental conditions, and genes: (**a**) bar plot, (**b**) individual lines plot and (**c**) stacked lines plot, are different modes of how Gene Expression Plotter (GXP) visualizes the expression profile of the example gene Solyc05g053550.3.1 (*CHALCONE SYNTHASE*). The three plots highlight how the expression of the example *CHALCONE SYNTHASE* responds to the experimental conditions. This *CHALCONE SYNTHASE’s* expression is up-regulated in *S. lycopersicum* but conversely not in *S. pennellii* following stress treatments of nitrogen deficiency (N-) and in combination with chilling temperatures (cold) and elevated light intensity (eL). Plot (**d**) compares the genetic response of this *CHALCONE SYNTHASE* with another gene of interest Solyc08g075570.4.1 (*UREA PROTON SYMPORTER)*. In contrast to the expression of *CHALCONE SYNTHASE*, gene expression of the *UREA PROTON SYMPORTER* is relatively low in both *S. pennellii* and *S. lycopersicum*.

With the aim of providing a single suite in which a user can visualize and analyze quantitative omics data and also share and publish the output, we programmed “Gene-Expression-Plotter” (GXP). GXP is available freely with a GPL license on GitHub: https://github.com/usadellab/GeneExpressionPlots (accession date 11 January 2022). GXP is provided with a comprehensive online documentation and a manual (see menu “Docs”). To use GXP, the user is first asked to import quantitative RNA-seq or metabolomics data in a tabular format. Each row should represent, e.g., a single quantified transcript. The transcript identifiers are typically provided in the first column. All following columns should contain the transcript quantifications for each respective genotype, replicate or treatment as specified by the column names (Figure 1a,b). GXP is made aware of the statistical factors differentiating in the experiment the respective biological replicates. This is encoded directly in the respective column names in the input quantification table. Here, as one studied factor the user can for example specify the time after an experimental treatment at which a sample was obtained or the type of stress treatment a sample was exposed to. Such a factor is then used by GXP to position points on the x-axis in plots. We consequently dub such factors “x-axis factors”. In the example data included in GXP, a column name positioning data points over the “ctrl1” x-axis tick (Figure 2) would be, for example, “S_lycopersicum.ctrl1.1” or “S_lycopersicum.ctrl1.2”. Note that the x-axis factor is identified by its location between the first and second “.” in the column name. The user can select another character, e.g., “*” instead of “.”. Strictly speaking, GXP accepts a single x-axis factor that can have multiple values. In the example data these can be “ctrl1” (control) and the stress treatments “cold” (chilling temperature), “eL” (extended light), and “N-” (nitrogen deficiency). Thus, x-axis factors join (in typical RNAseq experiments three) biological replicates that were subjected to the same experimental treatment into a single bin. Such x-axis bins are subsequently used to calculate y-axis error bars for data points representing several joined replicates (see Section 2.2 and Figure 2). Another type of factor can optionally be introduced and is used to compare for example biological species, genotypes, or different treatments. A good example for such a type of factor can be the comparison of a wild (*Solanum pennellii*) versus a domesticated (*S. lycopersicum*) tomato species. We dub such factors that group several biological conditions “group factors”. Note that these group factors are not used to generate tick labels on the x-axis, but rather imply two plots showing, e.g., gene expression in the wild type and domesticated species side-by-side (Figure 2a,b). A user can specify as many group factors as were investigated in a respective research project. Additionally, in the case of group factors, GXP is made aware of these simply through the column names of the counts table input. In the example data the column names “S_lycopersicum.ctrl1.1” and “S_pennellii.ctrl1.3” indicate the group factor “species” with values *S. lycopersicum* and *S. pennellii*, respectively. Note that the group factors are also identified by their position in the column names, appearing before the x-axis factor and separated by the “.” character. As mentioned before, the user can specify any other character as separator, e.g., “*”.

Optionally, GXP can use any extra information associated with specific quantified transcripts or metabolites. This is done by loading a separate table in which each row corresponds to a single quantified entity (transcript or metabolite), and in which each column holds additional generic information (Figure 1c,d). Examples of such additional generic information include ontological annotations informing about the molecular function of proteins, e.g., terms from the Mapman4 framework [3,36], from the Gene Ontology (GO) project [5], or from KEGG pathways [37], or differential expression between contrasted conditions, e.g., cold stress treatment versus control conditions. Information about differential expression can be provided in the form of logarithmic fold change of gene expression and/or adjusted *p*-values used to identify significant changes in gene expression. In fact, in this optional information table, the user can provide any information in the form of columns that contain either free text, numerical values, e.g., chemical properties, such as hydrophobicity, of metabolites, or categorical annotations about molecular protein function, similar to the terms obtained from Mapman4 ontology [36], the GO [5], the KEGG [37], or InterPro [4]. Note that GXP offers tools to help the user obtain and import molecular gene function annotations in the form of Mapman Bins for his data in the respective “Mapman functional annotations” section of the “Tools” menu. Importantly numeric and categorical information can be used to carry out subsequent overrepresentation analysis, while all information is used in the gene browser to provide the user with a rich database about the studied transcripts or metabolites (Section 2.1.1 and Figure 1e). 

Gene Expression Plotter is freely available on GitHub for direct use (https://usadellab.github.io/GeneExpressionPlots; accession date 11 January 2022). It is provided with example RNAseq data from a study on stress response contrasting wild with domesticated tomato species [38]. In collaboration with one of the original authors, this data has been used to directly compare and benchmark the results produced by GXP with the already published findings. This example data can be loaded by clicking on “Load example data” in the “data” menu.

#### 2.1.1. Browsing and Searching Gene Information

After having loaded the input data, the user can browse gene information in a searchable interface and make this information available to all who have access (Figure 1e). The presented information includes the respective quantitative data extracted from the “quantifications table” (see Section 2.1) and any further information about the respective transcripts or metabolites extracted from the “information table” (see Section 2.1). A user can, e.g., search for a gene of interest “chalcone synthase” and inspect all transcripts associated with this molecular function, their respective expression, and additional information such as conserved protein domains.

#### 2.1.2. Saving Work and Exporting Data

At any stage of using GXP, the user can export and save all imported data, plots, and analyses by using the dedicated “Export GXP Database” functionality (Figure 1f). The generated database can be used later to resume previous work or share plots and results. The exported GXP database contains a configuration file (“GXP_settings.json”, see manual for more details) that can be used, e.g., to change the order of “x-axis factors”, the unit of the expression values, and the various field separators used to load the tables into the application. At any stage all data is strictly kept on the user’s computer and at no point is user data sent through the web.

### 2.2. Visualizing Quantitative Data

The user can generate plots showing the expression of individually selected genes. Available visualizations are bar- (Figure 2a) and line-plots (Figure 2b). In these plots, “x-axis factors” (see Section 2.1) define the position of points on the x-axis. If the user has provided “group factor” information (see Section 2.1) that further groups biological replicates, e.g., species, genotype, or different treatments. This information will be visualized by the color or position of plotted values (Figure 2; domesticated tomato *S. lycopersicum* in red on the left side and wild *S. pennellii* in green on the right side). Quantifications differing between group factors, e.g., domesticated versus wild tomato species, can either be plotted in two graphs side-by-side (Figure 2a,b) or in a single graph as differentially colored stacked curves (Figure 2c). The user can also visualize the expression of multiple genes/metabolites in the same plot using all mentioned types: bar, single, or stacked curves (Figure 2d for an example with bars). In this case, as mentioned, the color distinguishes genes while the group factors are shown in their separate graphs side-by-side. All plots are interactive in that upon hovering over data points with the mouse, the user is presented with the respective values in a little overlay window. Hovering with the mouse over a specific point will display the underlying plotted data corresponding to that point, or bar, respectively. All plots a user generates can be saved and downloaded in high quality scalable vector graphics and used for publication purposes. Furthermore, exporting the GXP data and state optionally saves generated plots and analyses as well (see Section 2.1).

### 2.3. Assessing Similarity of Biological Replicates Based on Either Gene Expression or Quantified Metabolites

Typically, during the analysis and interpretation or RNAseq or metabolomics experiment results, one wants to distinguish within, i.e., biological background noise, from between-group differences to enable drawing significant conclusions in terms of an organism’s response to contrasting experimental conditions. Only if the in-between-group variation is not silenced by the background noise, can the data be used to elucidate the original biological questions motivating the study. To assess background noise and in-between-group variation, typically several (at least three in RNAseq experiments) biological replicates sharing the same experimental condition are quantified. Subsequently, similarity of the quantified replicates should be recognizable over the background noise to indicate that the quantifications can be used for the investigation of the original biological question motivating the study. This similarity can be assessed and visualized using correlation or Euclidean distance based hierarchical clustering and principal component analysis. 

#### 2.3.1. Hierarchical Cluster Analysis

**Figure 3:**
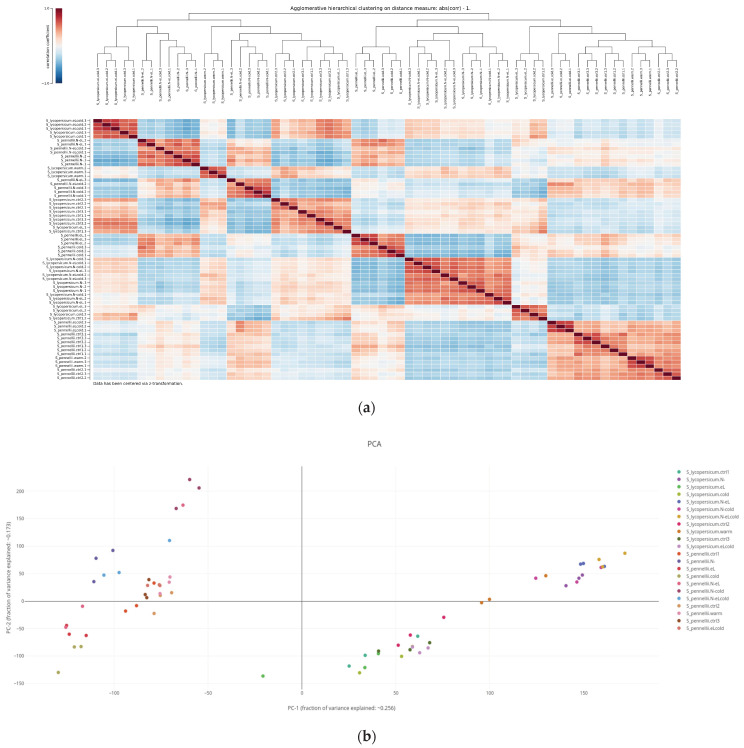
Screenshots of plots investigating likeliness of gene expression assessed in different biological replicates. Plot (**a**) shows the result of correlation-based hierarchical clustering in the form of a dendrogram and a correlation heatmap. In the top left corner, a scale color-codes the calculated correlation coefficients. In the top middle, the dendrogram represents the result of hierarchical clustering of all loaded biological replicates. In this example, the plot informs the user of their choice to z-transform the data before the calculation of correlation (lower left corner). Upon hovering with the mouse over single cells of the correlation matrix, the user is presented with the respective correlation value between the two biological replicates represented by the cell’s row and column, respectively. This example shows how Gene Expression Plotter (GXP) helps the user to assess how well the applied experimental conditions and treatments are reflected in terms of quantified gene expression. Here, serving as a quality check, the statistical factors “species” and “stress treatments” mostly imply the grouping of biological replicates, highlighting that the experimental setup and bioinformatics analysis yielded data fit to carry out the original biological question of the study, namely to elucidate the genetic responses to the applied stress treatments and subsequently compare these genetic responses between the two studied species of tomato. A plot highlighting similar patterns is shown in (**b**). Here, a principal component analysis (PCA) has been carried out on z-transformed data. The resulting scatter plot of the two most important principal components (PC) confirms that the color-coded biological replicates (legend in the top right corner) mostly group by the factors “species” and “stress treatment”, i.e., are found in close proximity within the scatter plot. When hovering with the mouse over single data points, the user is presented with the exact PC values and the name of the respective biological replicate represented by the data point. Using the axes labels, the user is informed about how much of the observed variation is explained by the two respective principal components PC1 (here: approx 25.6 %) and PC2 (here: approx. 17.3 %). As in (**a**), the PCA and resulting scatter plot indicate that biological replicates group well together, implying that within this example study, the influence of treatment and genotype on gene expression is well distinguishable from the biological background noise.

Results of a hierarchical cluster analysis, executed on optionally z-transformed values, are visualized in a heatmap whose axis is accompanied with a tree dendrogram representing the hierarchical clusters that the respective biological replicates have been grouped into (Figure 3a). A transposed cluster analysis can also be carried out, in which all or a selected subset of transcripts/metabolites are grouped by similarity of their respective quantitative data. GXP computes and visualizes hierarchical cluster analysis on demand in the user’s browser. The user can either select correlation or Euclidean distance between replicates’ gene expression vectors as a basis for the clustering analysis (see Section 4.3 for more details). The potentially demanding analysis is carried out in the background, using so called “web-workers”, and therefore does not block the user interface. While an analysis runs, a plot showing a loading icon is immediately created, indicating ongoing calculation. Once complete, the plot color indicates either the correlation values or euclidean distances between respective replicates, depending on the user’s original choice. A color scale is provided, and the plot is interactive, in that hovering with the mouse over a heatmap cell will display the calculated likeness value assessed for the respective pair the cell’s row and column corresponds to, respectively.

#### 2.3.2. Principal Component Analysis

Results of principal component analysis (PCA) are typically visualized as a subclass of scatter-plots where the two axes represent the two principal components that explain most of the observed variance between samples. GXP carries out a PCA on user demand and runs the respective calculation in the user’s browser in the background, thus not blocking the user interface. Once the calculation is finished, the loading icon indicating ongoing processing disappears and the respective scatter plot becomes visible (Figure 3b). Biological replicates are color coded so that replicates that have identical x-axis factors (Section 2.1), e.g., all replicates belonging to the wild tomato species *S. pennellii* that also have been exposed to the same cold stress conditions receive the same color. In the PCA scatter plot hovering with the mouse over a data point will display the name of the underlying biological replicate and the values of the two visualized principal components interactively.

### 2.4. Mapman Web Browser Plots

**Figure 4:**
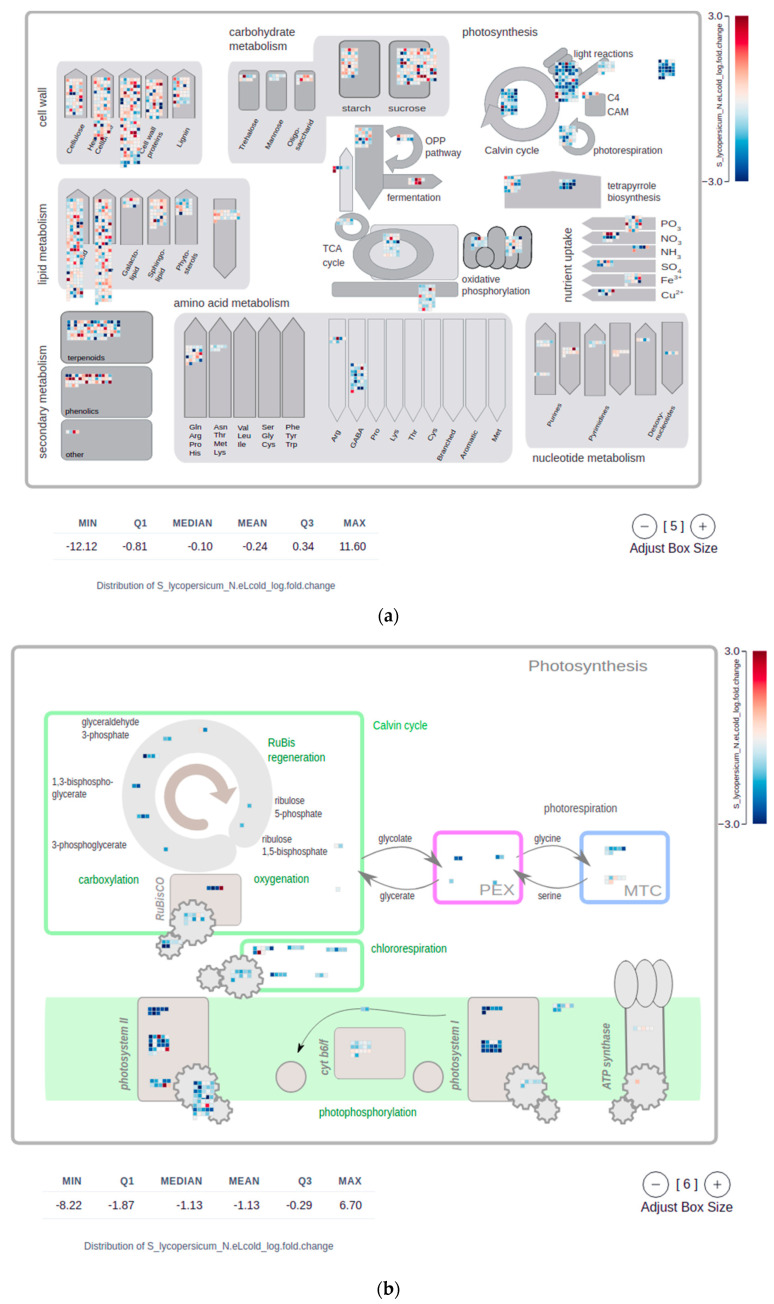

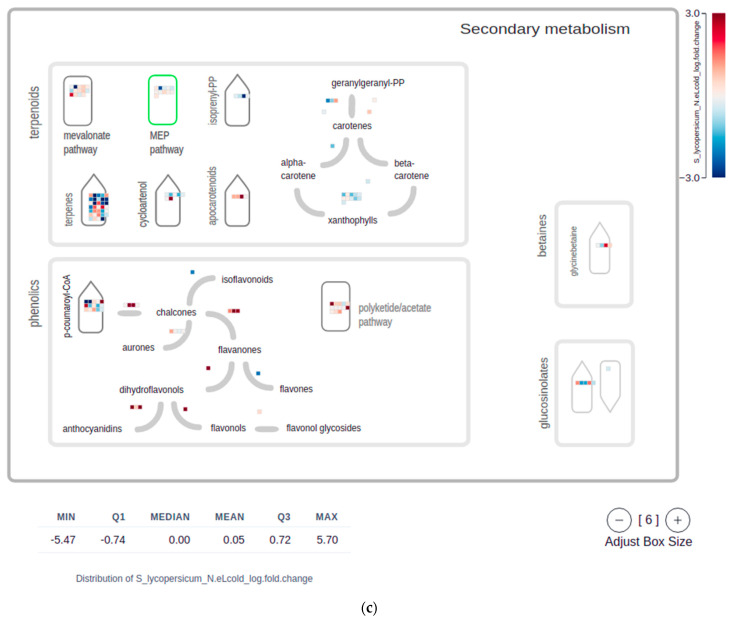
Screenshots of Mapman plots [1,2] used to visualize the genetic response to experimental stimuli in the form of metabolic sketches. Genes are mapped to areas in the sketches according to their molecular function. This gene function is directly extracted from the respective Mapman Bins [36] the genes are assigned to [3]. Each gene is represented by a single-colored box, where the color represents a numeric value, in this example the logarithmic fold change of gene expression (log-FC) between control and stress treatment. A legend in the top-right corner informs about the color-scale used to represent these numeric values. At the bottom of each Mapman plot, a summary statistic informs the user about the distribution of the respective numerical values, here the log-FC, shown in the plot. An interactive control in the bottom-right corner allows the user to adjust the sizes of the boxes, each representing one gene. Plot (**a**) shows a metabolic overview sketch and highlights how in the example data the expression of genes associated with photosynthesis is down regulated in *S. lycopersicum* following stress treatments (blue boxes in the respective top-right corner matrices). This down-regulation particularly affects genes of the light reaction, calvin cycle, and photorespiration pathways. Plot (**b**) sheds more light on this genetic response and zooms into the effect of stress treatments on the expression of genes associated with Photosystem I and II. Another detailed representation of the observed genetic response to stress treatment is shown in plot (**c**), elucidating how the expression of genes involved in terpene and carotene synthesis is down-regulated in *S. lycopersicum*.

The Mapman frameworks (Mapman4 [36] and the older version Mapman v.3.6 [39]) comprise a manually curated vocabulary (ontology) to describe the function of land plant proteins. Mercator and Mercator4 [3,36] are efficient and accurate genome scale annotation pipelines that assign the descriptions of Mapman v.3.6 and of Mapman4, respectively, to query proteins or transcripts. The desktop application MapMan [1,3,36,39]) has been developed to visualize annotations of the Mapman frameworks in the context of gene expression data. Based on a proof-of-principle code [1,40] we also developed a simple MapMan web browser application. The same as in the MapMan desktop application, a user can choose one of several basic metabolic cellular sketches, e.g., “Metabolism overview”, “Photosynthesis”, or “Secondary metabolism” (Figure 4). In these sketches, small squares represent proteins or transcripts with functional annotations semantically corresponding to that region in the diagram. For example, all proteins with functions related to the Calvin cycle would appear as small boxes in the upper left area of the “Photosynthesis” sketch (Figure 4b). 

The user chooses either a group of expression values for biological replicates belonging to the same experimental condition (x-axis factor; see Section 2.1), or any arbitrary numerical information provided in the optional information table. This can be the logarithmic expression fold change as typically shown in pathway diagrams comparing two experimental conditions, e.g., control versus cold stress. Instead of using logarithmic fold change values as a measure of the intensity of a transcriptional response under different experimental conditions, the user can also choose adjusted *p*-values produced by differential gene expression analyses. Finally, the user can choose how the color gradient is dispersed over the selected numerical values. The choice is between a divergent scale from a fixed negative value to the positive counterpart (as in the MapMan desktop application which focuses on log fold change data), or a continuous scale ranging from zero, or the first quartile, to the third quartile. The MapMan web browser plots are interactive, hovering with the mouse over a specific box displays the gene identifier, the Mapman4 protein description, and the numerical information assigned to the gene. 

### 2.5. Overrepresentation (Enrichment) Analysis

**Figure 5:**
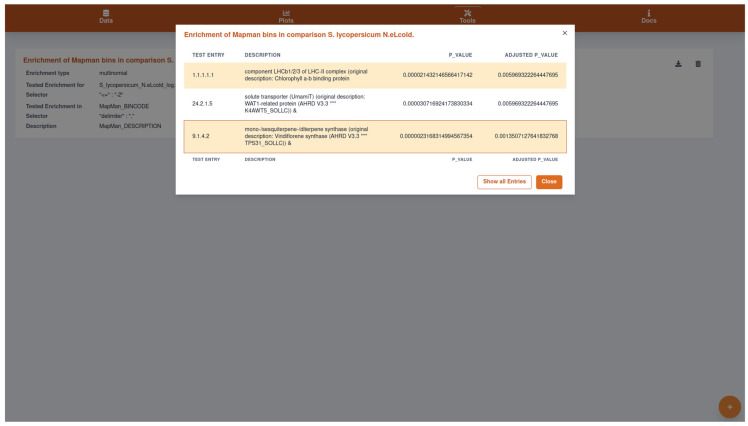
Screenshot of the result of an enrichment analysis (EA) carried out on the example data. This analysis is available in the “Tools” menu (screwdriver and wrench icon in the top panel). In the lightly faded-out background, the carried-out enrichment analysis can be seen. If more such analyses were done by clicking on the round plus icon in the bottom-right corner, they would also appear in this list. Clicking on the respective analysis opens the table shown in the highlighted foreground overlay. In it, the user is presented with significant results, while the button in the bottom-right corner “Show all Entries” enables the inspection of all, not only the annotations significantly tested for overrepresentation. In the shown example, the EA identified molecular gene functions overrepresented among genes whose expression is down-regulated in *S. lycopersicum* in response to the applied stress treatments, a combination of nitrogen deficiency (N-), chilling temperatures (cold), and elevated light intensity (eL). In this case, the results support the observation made for the example data earlier in Figure 4a,b, i.e. the response to stress treatments in the form of down-regulation of genes associated with (i) photosynthesis and (ii) terpene and carotene biosynthesis. Among the down-regulated genes, the molecular functions (i) “Chlorophyll a-b binding protein” in the “LHC-II complex” (Mapman Bin 1.1.1.1.1) and (ii) “UmamiT solute transporter” (Mapman Bin 24.2.1.5), a “sesquiterpene synthase”, and “diterpene synthase” (Mapman Bin 9.1.4.2) are significantly overrepresented (all adjusted *p*-values < 0.006). Thus the Mapman plots and enrichment analyses truly help to elucidate the genetic response in *S. lycopersicum* to the stress treatments applied in the example study.

To qualitatively describe a biological response, often, annotations about biological processes and molecular functions are analyzed. Those annotations found to be significantly overrepresented among selected genes or metabolites of interest characterize that group of genes. The selection criteria can, e.g., be significant up- or down-regulation of gene expression contrasting two experimental conditions, e.g., control versus cold stress treatment. Typically, Fisher’s exact test is used to determine whether the number of annotations within the selected genes significantly deviates from the number of annotations found within the background, i.e., the whole genome or metabolome. GXP offers the user an easy way to carry out such overrepresentation analysis (ORAs). The user specifies a criterion shared by all selected quantified entities, i.e., either transcripts or metabolites, and additionally selects annotation terms for which overrepresentation should be tested. Alternatively, the user can select the transcripts or metabolites of interest manually by entering their respective identifiers. It is noteworthy that GXP is agnostic to the underlying data structure, consequently the user can use any information originally loaded with the “information table” (Section 2.1). In principle, ORAs can be carried out for metabolite data, even though these analyses are less common for targeted metabolomics studies. Consequently, a great variety of enrichment analyses can be carried out. In this, each single calculation of Fisher’s exact test produces a corresponding single *p*-value, i.e., one *p*-value for each tested annotation. Currently, these *p*-values are corrected for multiple hypothesis testing using Bonferroni’s or the Benjamini-Hochberg method. All calculations are carried out in the background, so that user experience is not interrupted. The final result is a table in which for each tested annotation the corresponding adjusted *p*-value is shown (Figure 5). These results can be exported along with the data and plots by clicking the “Export GXP Database” button in the “Data” submenu (see Section 2.1). 

### 2.6. Usage of GXP to Publish Data along with Plots and Analysis Results

As previously mentioned, a user can save a GXP work-session by exporting all data, plots, and analysis results into a downloadable “GXP Database”. Such a GXP Database file can be made publicly available, e.g., by uploading it to a web-server such as GitHub or by providing it in the form of a supplement to a scientific article. Readers can thus download the published GXP Database, load it into GXP and explore the data, plots, and analysis results restored from the original work session. 

Another, more luxurious, mode of publishing data, plots, and analysis results along with GXP is included in the GXP manual. A user can deploy a copy of GXP together with an exported database to a dedicated webserver. GitHub-pages offers this option free of charge. This creates a link that can be cited in upcoming publications, does not require any maintenance work, and will be functioning as long as GitHub is maintained. A comprehensive (seven steps) step-by-step guide to set up such a tailored copy of GXP with specific user data, plots, and analysis results has been included in the online manual.

Thus, by using either of the above two methods, not only the raw expression counts and differential expression analysis result data could be provided, but also pregenerated supplemental plots highlighting the scientific results could be discussed in publications. This makes the data free to be explored by third parties in their own context of interest, possibly reaching beyond the scope of the publication.

## 3. Discussion

The availability, efficiency, and relatively low cost of next-generation sequencing and metabolomics technologies allows their application in a wide variety of plant science research projects. Quantification of gene expression or metabolites and contrasting these quantifications between different experimental conditions is implemented in many standard pipelines. However, the need for simple visualization, summarization, and further selected analyses revealing similarity between biological replicates and overrepresented molecular functions in sets of selected transcripts or metabolites is key for biological interpretation of these datasets. We revised platforms and software solutions that have been developed to provide the user with tools to quantify RNAseq raw data and contrast this quantified data in differential expression analysis. The revision includes tools that generate scientific plots, carry out clustering, principal component, and overrepresentation analysis [1,3,6,7,8,9,10,11,12,13,14,15,16,17,18,19,20,21,22,23,24,25,26,27,28,31,32,35,41]. However, the presented tools require either some programming expertise, or manual installation of software, or send potentially confidential data via the web to dedicated servers. Spreadsheet applications are often used to partially fill this gap, but the resulting plots are not interactive, and spreadsheet programs do not easily allow clustering, principal component, or overexpression analyses. In this context, we introduced Gene Expression Plotter (GXP) that provides the user with the means to load, analyze, and visualize quantitative and qualitative Omics data in the browser without the need for programming expertise or software installation. Additionally, GXP does all calculations locally in the browser without any need to submit data to servers. We incorporated into GXP the first mature and no-installation-required version of the popular Mapman tool [1,2]. This enables the user to summarize, in high quality plots, the gene functions, particularly up- and down-regulation, in a genetic response to experimental treatments, e.g., contrasting control and cold stress treatments. Hence, GXP offers simple solutions to explore and analyze Omics data and to generate publication grade plots. We furthermore explained how GXP can be used to publish Omics data along with plots and analysis results either by simply providing the community with a ready to use GXP database file, e.g., in the form of an article supplement, or by setting up an online copy of GXP already including the mentioned data, plots, and results. In this, the latter method can be done directly on GitHub free of charge by following seven simple steps that only require a web browser and a GitHub account.

To help the reader and user explore the value of using GXP and to benchmark GXP’s functions, we included real research data from a published study on stress response in two tomato species [38]. We verified with the aid of one of the authors of this original study that (i) GXP reproduces and visualizes the already published findings, (ii) aids in the exploration of Omics data and promotes the formation of scientific hypotheses (Figure 1, Figure 2, Figure 3, Figure 4 and Figure 5), and (iii) thus helps to elucidate, e.g., the genetic response to experimental stimuli, i.e., the original biological question motivating the study. 

GXP is open-source software, runs entirely in the web browser and all code is automatically unit-tested, and thus is ensured to carry out correct calculations. Additionally, all code has been written adhering to current cutting-edge coding standards. Importantly, GXP is versatile in terms of its input. GXP consumes data about quantified entities, typically transcripts or chemical compounds (metabolites). Optionally, the user can supply further arbitrary either free text, categorical, and/or numerical information about the quantified entities and use, especially the latter two types of input data in GXP’s plots and analyses. This generic approach to quantitative data visualization, exploration, and analysis, along with the option to easily setup a copy of GXP with specific user data, plots, and analysis results, specifically qualifies the GXP code base for reuse and extension in the typical open-source community approach. We indeed believe that GXP can become a platform for which over time, more and more functions, plots, and analyzes can be provided by third party developers.

## 4. Materials and Methods

Gene-Expression-Plotter (GXP) was implemented in TypeScript (version 4.5.2; https://www.typescriptlang.org/; access date 11 January 2022) as a standalone application (single page application; SPA) executed in the web-browser. This form of implementation implies that even though the SPA is obtained from a webserver, after opening it in the browser no data is ever sent to any webserver for analysis. All calculations, plots, and analysis are carried out right on the user’s computer in the browser itself; thus, data confidentiality is guaranteed. For implementation, the ReactJS (version 17.0.1; https://reactjs.org/; access date 11 January 2022) and Chakra UI (version 1.7.2; https://chakra-ui.com/; access date 11 January 2022) libraries were used to build the user interface. The ViteJS library (version 1.3.6; https://vitejs.dev/; access date 11 January 2022) was used for tooling. GXP can be accessed on GitHub pages (https://usadellab.github.io/GeneExpressionPlots; access date 11 January 2022); every time a new version is pushed to GitHub, the new code is compiled and automatically deployed to GitHub pages using GitHub actions. GXP’s source code is freely available on GitHub (https://github.com/usadellab/GeneExpressionPlots; access date 11 January 2022) under a GPL-3 license.

### 4.1. Input and Output Data

Expression or metabolite data, and additional information about transcripts or metabolites, can be loaded into GXP (see Section 2.1). Alternatively, a previous work-session can be restored using the “Import GXP Database” function in the “Data” menu (see Section 2.1). All data is stored in memory, no data is ever sent via the internet to any backend server. Memory state management has been implemented using the MobX library (version 6.3.7; https://mobx.js.org; access date 11 January 2022).

### 4.2. Gene Expression Plots

All introduced plots (see Section 2.2) were implemented with the plotly.js Javascript library (version 2.6.3; https://plotly.com/javascript/; access date 11 January 2022). Plot data and definitions are stored in memory and thus can be exported to and restored from GXP Databases (see Section 2.1.2).

### 4.3. Hierarchical Cluster Analysis

Similarity between biological replicates is either assessed using correlation or Euclidean distance between the respective gene expression vectors (see Section 2.3.1). In this, correlation values *c_i_*_,*k*_ are transformed to distance values *d_i_*_,*k*_ as follows:

*d_i_*_,*k*_ = 1 − abs(*c_i_*_,*k*_),

with “abs” returning the absolute value of its real number argument.

Thus, complete anticorrelation as well as complete correlation are interpreted as maximum likeliness of numeric quantification vectors. 

Euclidean distance measures are computed with the ml-distance Javascript library (version 3.0.0; https://github.com/mljs; access date 11 January 2022). Hierarchical clusters are identified using the ml-hclust library (version 3.1.0; https://github.com/mljs; access date 11 January 2022). The heatmap and the respective dendrogram visualizing the results of hierarchical clustering are plotted with the visx (version 2.4.0; https://github.com/airbnb/visx; access date 11 January 2022) library that incorporates the popular and well proven D3 library (version 7.1.1; https://d3js.org/; access date 11 January 2022into React.js.

### 4.4. Principal Component Analysis (PCA)

In GXP, a PCA can be carried out to identify and visualize likeliness between gene expression or metabolite concentrations of biological replicates (see Section 2.3.2). The principal component analysis is computed with the help of the ml-pca library (version 4.0.2; https://github.com/mljs; access date 11 January 2022). In this calculation, all data points are considered. The respective plot visualizing the first two principal components contributing most to the observed differences is created with plotly.js (version 2.6.3; https://plotly.com/javascript/; access date 11 January 2022).

### 4.5. MapMan Visualizations

GXP offers to summarize genetic expression or responses to contrasting experimental conditions in the form of Mapman plots [1] (see Section 2.4). All available canvas sketches (version X4.3) upon which to draw boxes to represent transcripts’ quantification values were downloaded from the respective “MapMan Store” online repository (version X4.3; https://mapman.gabipd.org/mapmanstore; access date 11 January 2022) and included in the GXP package. Based on the proof-of-concept implementation [40], the visualization was programmed with the D3 library (version 7.1.1; https://github.com/d3/d3; access date 11 January 2022).

### 4.6. Overrepresentation Analysis

Gene Expression Plotter offers the user the ability to define sets of transcripts (genes) or sets of metabolites of interest, either by stating the respective identifiers one-by-one or by defining a selection criterion (see Section 2.5). Subsequently, annotations assigned to the selected genes are tested for being over-presented in comparison to the background, which is the whole information table, i.e., the genome or the metabolome (see Section 2.1). Each of these tests is carried out as Fisher’s exact test resulting in a single *p*-value indicating how likely the observed annotation numbers can be explained by the null hypothesis, i.e., variations of the background annotations. In Fisher’s exact test contingency tables are created and *p*-values calculated using the hypergeometric probability distribution (HGD) [42]. The calculation of specific HGD *p*-values is carried out with the GNU scientific library (version 2.6; git://git.savannah.gnu.org/gsl.git; accessed on 11 January 2022) [43] which was compiled to web-assembly (version 1.0; https://webassembly.org/; access date 11 January 2022) for usage in the web-browser with Javascript. This compilation was done with emscripten (version 2.0.25; https://emscripten.org/; access date 11 January 2022). To calculate the likelihood of the alternative hypothesis that the observed numbers of annotations are not just as is in the contingency table but potentially greater, i.e., more extreme, more contingency tables of more extreme distributions are created and tested, respectively. Resulting *p*-values are summed up until no more extreme contingency tables can be generated, i.e., the respective cells contain zero. This procedure has been implemented in Javascript and correctness of the calculations is confirmed by dedicated automatic unit software tests.

### 4.7. Example Dataset

To demonstrate GXP’s qualities, published data sets from two tomato species were used [38]. In brief, two tomato species (*S. lycopersicum* and *S. pennellii*) were grown in rockwool blocks and watered with water for 16 days. Afterwards, the seedlings were fertilized with half-strength Hoagland solution for 14 days, followed by full-strength Hoagland solution (5 mM KNO3, 5 mM Ca(NO3)2, 2 mM MgSO4, 1 mM KH2PO4, 90 μM FeEDTA, plus micronutrients) for a further 11 days. A total of 6 weeks after germination, plants were stressed by nitrogen deficiency (N-), chilling temperatures (cold), warmer temperature regime (warm), or elevated light intensity (eL) and combinations thereof (Ncold, N-eL, eLcold and N-eLcold). After 1 week of stress treatment, leaflets of the fourth leaf (counted from the tip) were sampled, immediately frozen in liquid nitrogen and stored at −80 °C. Total RNA was extracted and treated with DNase followed by mRNA enrichment, and subsequently analyzed using an Illumina-platform (HiSeq) sequencing 2 × 75 bp paired-end reads. 

Raw reads were trimmed using Trimmomatic [44]. An artificial transcriptome was built using default settings of StringTie [45,46] and back mapped to the genome of *S. lycopersicum* (version ITAG 4). Data analysis was performed using R (version 3.5.2) [29]. Read abundances were analyzed using R-packages limma [47], edgeR [48], and tximport [49].

### 4.8. Automated Software Tests Ensure Correctness of Implemented Analyses

The code written to carry out the z-transformation, correlation, clustering, principal component, Fisher’s exact test, overrepresentation, and *p*-value adjustment calculations provided within GXP are all verified for correctness using automated software, so called unit tests. These tests use data obtained from real life science projects and ensure that the respective functions behave correctly even in “edge cases” where data is unexpectedly abnorm, e.g., empty. These tests can be run automatically and thus ensure correctness of calculations even if future extensions are programmed. All tests are located in the cypress/tests/integration directory in GXP’s GitHub code repository. 

## Data Availability

All code, example and test data is available at https://github.com/usadellab/GeneExpressionPlots (accession date 11 January 2022).

## Supplementary Materials

The following supporting information can be downloaded at: https://www.mdpi.com/article/10.3390/plants11060745/s1, Text S1: Review of comparable tools to visualize and analyze RNAseq or Metabolomics quantitative data, Text S2: Table of used software packages and frameworks.
