# Supplementary material for: GXP: Analyze and Plot Plant Omics Data in Web Browsers
Source: Plants (Basel). 2022 Mar 11;11(6):745. doi: 10.3390/plants11060745 (PMC8952246; doi:10.3390/plants11060745)
Supplement: Supplementary file 1 [file plants-11-00745-s001.zip › GeneExpressssionPlotter_Paper-Supplemental_Text_S1.pdf]

GXP: Analyze and plot plant omics data in web browsers

Supplemental text S1

Review of comparable tools to visualize and analyze RNAseq or Metabolomics quantitative data

In this document we review the tools presented in the introduction section in order to explain the motivation that led to the development of Gene Expression Plotter (GXP) in the context of comparison with the mentioned tools. We briefly summarize their features, requirements, and technical traits. In this comparison, we focus on features that are key in the context of this article. Each tool has its section headed by the tool's name.

Abbreviations

ANOVA:	Analysis of variance
DE:	Differential Expression
DEG:	Differentially Expressed Gene
DEGES:	DEG Elimination Strategy
FC:	Fold-Changes
FDR:	False Discovery Rate
GSEA:	Gene Set Enrichment Analysis
GUI:	Graphical User Interface
KEGG:	Kyoto Encyclopedia of Genes and Genomes
MDS:	Multi-dimensional Scaling
ORA:	Over-Representation Analysis
PCs:	Principal Components
PCA:	Principal Component Analysis
PPI:	Protein-Protein Interaction
scRNA-Seq:	Single cell RNA sequencing
RLE:	Relative log expression
TCC:	Tag Count Comparison
TMM:	Trimmed mean of M values

PaintOmics3

- Hernández-de-Diego R, Tarazona S, Martínez-Mira C, Balzano-Nogueira L, Furió-Tarí P, Pappas GJ Jr, et al. PaintOmics 3: a web resource for the pathway analysis and visualization of multi-omics data. Nucleic Acids Res. 2018;46: W503–W509.

General

Requires installation?	No
Sends data through the web?	Yes
Can it be used to publish data?	An option to share results exists
Coding required?	No

Specific

- Specialized in visualization of omic data onto KEGG pathways.
- “Paintomics takes complete transcriptomics and metabolomics datasets, together with lists of the significant gene or metabolite changes, and paints this information on KEGG pathway maps.”

PlantReactome

- Naithani S, Gupta P, Preece J, D’Eustachio P, Elser JL, Garg P, et al. Plant Reactome: a knowledgebase and resource for comparative pathway analysis. Nucleic Acids Res. 2020;48: D1093–D1103

General

Requires installation?	No
Sends data through the web?	Yes
Can it be used to publish data?	An option to share results exists
Coding required?	No

Specific

- Uses knowledge of gene-orthology to project omics data of any plant species onto rice known pathways.
- Takes in protein-protein interaction data as input.

ePlant

- Waese J, Fan J, Pasha A, Yu H, Fucile G, Shi R, et al. ePlant: Visualizing and Exploring Multiple Levels of Data for Hypothesis Generation in Plant Biology. Plant Cell. 2017;29: 1806–1821.

General

Requires installation?	No
Sends data through the web?	No - data cannot be uploaded
Can it be used to publish data?	An URL can be generated to work done
Coding required?	No

Specific

- Integrates interactome, transcriptome, and 3D molecular structure data published for *Arabidopsis thaliana.*
- Allows exploration of already integrated data through multiple ways.
- Allows for hypothesis formation and testing.
- Can be deployed on any server.

SLEUTH

- Pimentel H, Bray NL, Puente S, Melsted P, Pachter L. Differential analysis of RNA-seq incorporating quantification uncertainty. Nat Methods. 2017;14: 687–690.

General

Requires installation?	Yes
Sends data through the web?	No
Can it be used to publish data?	No
Coding required?	Yes

Specific

- R/Shiny application.
- Implements an alternative method for the identification of differentially expressed genes.
- Implements a ready to use RNA-Seq pipeline that generates expression counts and differential expression results.

MVapp

- Julkowska MM, Saade S, Agarwal G, Gao G, Pailles Y, Morton M, et al. MVApp—Multivariate Analysis Application for Streamlined Data Analysis and Curation. Plant Physiology. 2019. pp. 1261–1276. doi:10.1104/pp.19.00235

General

Requires installation?	No (optional for self-hosted)
Sends data through the web?	Yes, but does not store data on the server
Can it be used to publish data?	Yes
Coding required?	No

Specific

- Carries out multivariate analyses to reveal genotype-to-phenotype relationships.
- Can be used for interactive data curation, clustering, and quantile regression.
- Phenotypic trait modelling by curve fitting.
- Highlights potential outlier samples.
- Tests for significantly varying phenotypes (ANOVA, Kruskal-Walis)
- Plots: Heatmap/hierarchical clustering, PCA.
- Performs estimation of heritability.

GLIMMA

- Su S, Law CW, Ah-Cann C, Asselin-Labat M-L, Blewitt ME, Ritchie ME. Glimma: interactive graphics for gene expression analysis. Bioinformatics. 2017;33: 2050–2052.

General

Requires installation?	Yes
Sends data through the web?	No
Can it be used to publish data?	Yes
Coding required?	Yes

Specific

- R package extending popular plots generated for results obtained from differential gene expression identification.

- Plots: multidimensional scaling plots and mean-difference plots.
- Individual data points can be explored interactively by hovering over them in the generated plots. The expression of a single gene is then shown in a new plot side by side.

TCC-GUI

- Su W, Sun J, Shimizu K, Kadota K. TCC-GUI: a Shiny-based application for differential expression analysis of RNA-Seq count data. BMC Res Notes. 2019;12: 133.

General

Requires installation?	Yes
Sends data through the web?	No
Can it be used to publish data?	Maybe
Coding required?	No

Specific

- A GUI frontend to the TCC Bioconductor package for identification of differential expression.
- Test server available online.
- Performs DEGES (DEG elimination strategy) to iteratively normalize expression counts after removing top-ranked (differential expression) genes.
- Implements analysis, interactive plots: MDS, PCA, clustering.
- Generates interactive linked plots, e.g. volcano plot, hovering over a data point opens expression level barplot for that gene.
- Differences in expression between groups can be displayed using MA plots.

iGEAK

- Choi K, Ratner N. iGEAK: an interactive gene expression analysis kit for seamless workflow using the R/shiny platform. BMC Genomics. 2019;20: 177.

General

Requires installation?	Yes
Sends data through the web?	No
Can it be used to publish data?	Maybe

Coding required?	No

Specific

- R/Shiny desktop application.
- Carries out the identification of differential expression genes.
- Implements: PCA, correlation-based heatmap/clustering, gene expression boxplots (one box per sample/group), PPI based on pre-existing ENSEMBL data, ORA based on Reactome pathways.
- Plots: volcano plot.
- Specializes in usage for animals, as it uses Gene-Symbols and the Reactome pathways for published data-sets on human and mouse experiments.

Shiny-Seq

- Sundararajan Z, Knoll R, Hombach P, Becker M, Schultze JL, Ulas T. Shiny-Seq: advanced guided transcriptome analysis. BMC Res Notes. 2019;12: 432.

General

Requires installation?	No
Sends data through the web?	Yes
Can it be used to publish data?	Maybe
Coding required?	No

Specific

- R/Shiny GUI to pipeline analysis.
- Takes in Kallisto output or a count table.
- Performs normalization with DESeq2.
- Implements batch effect analysis and PCA plot is used to show the effect of surrogate variables.
- Implements differential gene expression analysis and co-expression network analysis.
- Gene set enrichment analysis (GSEA) for clusters can be done in the context of humans, mice, and yeast.
- Transcription factor binding side overrepresentation analysis in the context of human, mouse data.
- Generated plots include MA, PCA, Fold-Change x FC, volcano, expression plot with baseline.

pcaExplorer

- Marini F, Binder H. pcaExplorer: an R/Bioconductor package for interacting with RNA-seq principal components. BMC Bioinformatics. 2019;20: 331.

General

Requires installation?	Yes
Sends data through the web?	No
Can it be used to publish data?	Yes
Coding required?	No

Specific

- R/Shiny application.
- Implements PCA, normalization with DESeq2, heatmaps, box-plots, functional interpretation of principal components.
- Functional annotation based on Gene Ontology database can be done on the PCs.
- Box plots of data grouped based on experimental variables and selected genes of interest can be generated and explored.
- HTML reports can be generated at any state displaying code and output.

Shiny-DEG

- Wang S, Zhang Y, Hu C, Zhang N, Gribskov M, Yang H. Shiny-DEG: A Web Application to Analyze and Visualize Differentially Expressed Genes in RNA-seq. Interdiscip Sci. 2020;12: 349–354.

General

Requires installation?	Yes
Sends data through the web?	No
Can it be used to publish data?	Yes
Coding required?	No

Specific

- R/Shiny application.
- Supports multi-factor design models. DEG across all conditions can be identified.

- Plots: Boxplot of gene expression, Volcano plots showing the up and down-regulated genes, heatmap of cluster analysis (using Euclidean, Manhattan or Pearson correlation), PCA plot of DEG.

GENAVi

- Reyes ALP, Silva TC, Coetzee SG, Plummer JT, Davis BD, Chen S, et al. GENAVi: a shiny web application for gene expression normalization, analysis and visualization. BMC Genomics. 2019;20: 745.

General

Requires installation?	No (optional for self-hosted)
Sends data through the web?	Yes
Can it be used to publish data?	Yes
Coding required?	No

Specific

- R/Shiny application hosted on the Shiny server.
- Used for normalization and differential analysis of human or mouse feature count data.
- Supports a range of normalization strategies: t-score like, logCPM-edgeR, vst-DESeq2 and rlog-DESeq2.
- Pots: Clustered heatmap based on Pearson distance, PCA, volcano plot.
- DEA can be performed integratively using the DESeq2 workflow.
- Gene set enrichment analysis can be performed.

RNfuzzyApp

- Haering M, Habermann BH. RNfuzzyApp: an R shiny RNA-seq data analysis app for visualisation, differential expression analysis, time-series clustering and enrichment analysis. F1000Res. 2021;10: 654.

General

Requires installation?	Yes
Sends data through the web?	No
Can it be used to publish data?	Yes

Coding required?	No

Specific

- R/Shiny application.
- Supports normalization of data using DESeq2, TMM, RLE or edgeR's upper-quantile.
- Implements DEA using DESeq2, edgeR or bayseq.
- Pairwise comparisons of different conditions and time points can be made.
- The following Interactive plots can be generated: hierarchical cluster heatmaps, MA, Volcano plots and PCA.
- Performs enrichment analysis using gprofiler2.
- Implements RNA-seq Time series analysis.

COEX-Seq

- Kim SC, Yu D, Cho SB. COEX-Seq: Convert a Variety of Measurements of Gene Expression in RNA-Seq. Genomics Inform. 2018;16: e36.

General

Requires installation?	Yes
Sends data through the web?	No
Can it be used to publish data?	No
Coding required?	No

Specific

- R/Shiny application.
- Converts gene expression measurement data formats that are used in various bioinformatics tools for RNA-Seq analysis.
- Generates a summary report of the converted data in form of boxplots.

iSeq

- Zhang C, Fan C, Gan J, Zhu P, Kong L, Li C. iSeq: Web-Based RNA-seq Data Analysis and Visualization. Methods Mol Biol. 2018;1754: 167–181.

General

Requires installation?	No
Sends data through the web?	Yes
Can it be used to publish data?	Yes
Coding required?	No

Specific

- R/Shiny application.
- Supports data normalization by size factor (using DESeq) and quantile (using preprocessCore R package).
- DEA can be done using DESeq2.
- Functional enrichment is implemented using DAVID and GOseq R package.
- iSeq incorporates a link to DAVID official site where a list of previously obtained up or down-regulated genes can be used for functional enrichment.
- Plots: Box plot, PCA, Hierarchical Clustering, Volcano plots, Heatmap.

OnestopRNAseq

- Li R, Hu K, Liu H, Green MR, Zhu LJ. OneStopRNAseq: A Web Application for Comprehensive and Efficient Analyses of RNA-Seq Data. Genes . 2020;11. doi:10.3390/genes11101165

General

Requires installation?	No
Sends data through the web?	Yes
Can it be used to publish data?	Yes
Coding required?	No

Specific

- Web application hosted by Apache server.
- MySQL is used as the backend and PHP is used for the presentation layer.
- Implements analysis using a pipeline in Snakemake workflow.
- Performs RNA-seq analysis such as raw read quality check, differential analysis of gene expression, exon usage, alternative splicing, transposable element expression, allele-specific gene expression quantification, and gene set enrichment analysis

- RNA-seq data analyses based on human, mouse, yeast, fruit fly, zebrafish, and worm genomes as the tool provides their reference genomes and annotations for the user to select.
- The user can request for a genome of interest to be added if not present.
- Integrates DEBrowser and Shiny-seq for DEA and interactive investigation of DE data.

WASP

- Hoek A, Maibach K, Özmen E, Vazquez-Armendariz AI, Mengel JP, Hain T, et al. WASP: a versatile, web-accessible single cell RNA-Seq processing platform. BMC Genomics. 2021;22: 195.

General

Requires installation?	Yes
Sends data through the web?	No
Can it be used to publish data?	Yes
Coding required?	No

Specific

- Snakemake automated preprocessing pipeline and R/Shiny application.
- Processes Drop-Seq-based scRNA-Seq data.
- Implements a pipeline in Snakemake workflow for data pre-processing. The results are fed to the R Shiny web application for gene expression matrix generations, post-processing and plot generation.
- The Shiny app component can also consume external data not generated by WASP.
- Designed for scRNA-seq data.

DEIVA

- Harshbarger J, Kratz A, Carninci P. DEIVA: a web application for interactive visual analysis of differential gene expression profiles. BMC Genomics. 2017;18: 47.

General

Requires installation?	Yes
Sends data through the web?	No

Can it be used to publish data?	Yes
Coding required?	No

Specific

- R/Shiny application.
- Single page web application.
- Consumes results of DEA statistical test results as input.
- Provide an interactive interface for the exploration of differential expression data.
- Summarizes statistical test results of DEA.
- Gene identification is implemented in an interactive approach.
- Generates MA plot and volcano plot.

RNASeqGUI

- Russo F, Angelini C. RNASeqGUI: a GUI for analysing RNA-Seq data. Bioinformatics. 2014;30: 2514–2516.

General

Requires installation?	Yes
Sends data through the web?	No
Can it be used to publish data?	Yes
Coding required?	No

Specific

- R-Tcl/Tk application.
- Can be used to explore bam files, counting mapped reads against an annotation file, identification of differential expression genes using DESeq, DESeq2, EdgeR, NOISeq, BaySeq.
- Plots: Fold change plots, false discovery rate (FDR) histograms, p-value histograms and volcano plot, heatmap and PCA plot.

START App

- Nelson JW, Sklenar J, Barnes AP, Minnier J. The START App: a web-based RNAseq analysis and visualization resource. Bioinformatics. 2017;33: 447–449.

General

Requires installation?	Yes
Sends data through the web?	No
Can it be used to publish data?	Yes
Coding required?	No

Specific

- R/Shiny application.
- Performs normalization and regression of raw gene expression count data.
- Plots: PCA plot, heatmap based on euclidean distance, volcano plot, scatter, gene expression boxplot, analysis plot (using p-values and fold change).

DEApp

- Li Y, Andrade J. DEApp: an interactive web interface for differential expression analysis of next generation sequence data. Source Code Biol Med. 2017;12: 2.

General

Requires installation?	No
Sends data through the web?	Yes
Can it be used to publish data?	Yes
Coding required?	No

Specific

- R/Shiny application.
- Takes in raw count table (output of transcript quantification tools) and meta-data table.
- Genetic features with low counts can be filtered out within the tool’s data panel.
- Identifies differentially expressed genes using edgeR, DESeq-2 or limma-voom.
- Outputs of the different methods can be compared directly from within the interface.
- Plots: multidimensional scaling (MDS) plot, volcano plot, dispersion plot.

Plant Physiospace

- Hadizadeh Esfahani A, Maß J, Hallab A, Schuldt BM, Nevarez D, Usadel B, et al. Plant PhysioSpace: a robust tool to compare stress response across plant species. Plant Physiol. 2021;187: 1795–1811.

General

Requires installation?	Yes
Sends data through the web?	No
Can it be used to publish data?	No
Coding required?	depends

Specific

- R package provided along with a GUI written in R/Shiny
- A specialized algorithm compares changes in gene expression with curated signatures obtained from earlier published studies. Alike signatures are robustly identified and thus similar genetic responses to potentially very different stimuli or treatments can be identified. The tool enables the user also to compare two or more of her/his own differential expression profiles stemming from separate contrasted treatments.
- To get full access to the implemented functions a user needs to do some manual coding in R
- The core functionality is exposed through a R/Shiny interface

MeV / WebMeV

- Howe E, Holton K, Nair S, Schlauch D, Sinha R, Quackenbush J. MeV: MultiExperiment Viewer. Biomedical Informatics for Cancer Research. 2010. pp. 267–277. doi:10.1007/978-1-4419-5714-6_15
- Howe E, Holton K, Nair S, Schlauch D, Sinha R, Quackenbush J. WebMeV: MultiExperiment Viewer. Available: https://webmev.tm4.org/#/about

General

Requires installation?	Yes (MeV), No (WebMeV)
Sends data through the web?	No (MeV), Yes (WebMeV)
Can it be used to publish data?	No (MeV), Yes (WebMeV)
Coding required?	No

Specific

- MeV, multi experiment viewer, is a software suite to analyze large genomic data, with a particular focus on RNAseq.
- For those, who do not want to install it locally, there is a web-frontend available (WebMeV) which runs the analyses in a dedicated cloud.
- Implemented functions enable the user to load quantified expression data, provide metadata to assign biological replicates to experimental conditions or treatments, carry out differential expression analysis, cluster the biological replicates by several algorithms, including principal component analysis, and identify overrepresented gene functions (Gene Ontology terms), carry out a metabolic pathway analysis and identify modules of co-expressed genes.
- Gene expression can be visualized with boxplots and clustering results in heatmap plots.
