# Supplementary material for: GXP: Analyze and Plot Plant Omics Data in Web Browsers
Source: Plants (Basel). 2022 Mar 11;11(6):745. doi: 10.3390/plants11060745 (PMC8952246; doi:10.3390/plants11060745)
Supplement: Supplementary file 1 [file plants-11-00745-s001.zip › GeneExpressssionPlotter_Paper-Supplemental_Text_S2.pdf]

GXP: Analyze and plot plant omics data in web browsers

Supplemental text S2

Table of used software packages and frameworks

This supplement provides a summarizing table about software packages, modules, and frameworks used to implement GXP and its functions. The interested reader can thus quickly assess the software dependencies of GXP.

Table S2.1: List of packages, modules, or frameworks used to implement Gene-Expression-Plotter (GXP). The respective entries are listed by order of appearance in the main text.

Package, module, or framework	Version	Link
typescript	4.5.2	https://www.typescriptlang.org/
ReactJS	17.0.1	https://reactjs.org/
Chakra UI	1.7.2	https://chakra-ui.com/
ViteJS	1.3.6	https://vitejs.dev/
MobX	6.3.7	https://mobx.js.org/
plotly.js	2.6.3	https://plotly.com/javascript/
ml-distance	3.0.0	https://github.com/mljs/
ml-hclust	3.1.0	https://github.com/mljs/
visx	2.4.0	https://github.com/airbnb/visx/
D3	7.1.1	https://d3js.org/
ml-pca	4.0.2	https://github.com/mljs/
MapMan Store	X4.3	https://mapman.gabipd.org/mapmanstore/
GNU Scientific Library	2.6	git://git.savannah.gnu.org/gsl.git/
web-assembly	1.0	https://webassembly.org/
emscripten	2.0.25	https://emscripten.org/
